# 

**Published:** 1902-10-11

**Authors:** 


### NOTICE TO CORRESPONDENTS.

All MSS., Letters, Books for Review, and other matters intended for the Editor should be addressed The Editor, "The Hospital," The Hospital Building, 28 & 29 Southampton Street, Strand, W.C.
The Editor cannot undertake to return rejected MS., even when accompanied by stamped directed envelope.

**Notice to Advertisers and Subscribers.**

All Advertisements, Orders for Copies of The Hospital, and Business Communications should be addressed to **THE MANAGER, (Not to the Editor)**, The Hospital, 28 & 29 Southampton Street, Strand, London, W.C.
The Cost of Subscription, post free, is as follows.—Per week, 2½d.; per quarter, 2s. 8d.; per half-year, 5s. 3d.; per annum, 10s. 6d. Foreign:—Per annum, 15s. 2d., payable, in all cases, in advance.

**NOTICE.—Vol. XXXII. of "The Hospital" (April to September 1902) in handsome cloth binding will shortly be on sale at the office of this Journal, price, 7s. 6d. Cases for binding the Numbers contained in this Volume 1s. 6d. Index to Vol. XXXII., 2½d., post free.**

**The Monthly Part for October is now ready. Price 6d., by post 8d.**

### BOOKS RECEIVED.

William Hodge and Co., Glasgow.

"Annual Report of the Medical Officers of Health to the County Councils of Dunbarton and of Stirling." By John C. McVail, M.D., D.P.H., F.R.S.E.

William Pile, Wallington.

"Annual Report of the Medical Officer of Health of the Croydon Rural District."

Cassell and Company.

"A Manual of Medical Treatment or Clinical Therapeutics." By I. Burney Yeo, M.D., F.R.C.P. New and revised edition, in two volumes.

The Scientific Press.

"Ophthalmic Nursing." By Sydney Stephenson, M.B., C.M., F.R.C.S.E.

Longmans, Green and Co.

"The Practitioner's Guide." By Walter Carr, M.D.Lond. F.R.C.P.; T. Pickering Pick, F.R.C.S.; Alban H. G. Doran, F.R.C.S.; and Andrew Duncan, M.D., B.S.Lond., F.R.C.S., M.R.C.P.

Merser and Sons.

"Report on the Epidemic of Small-pox, 1901–2, in the Borough of Lambeth." By Joseph Priestley, B.A.Lond., M.D., D.P.H.

Wills, Limited.

"Nursing Homes, a Warning." By Sir Robert Harvey, D.L., J.P.

"The Zenana Bible and Medical Mission." The Zenana, Vol. ix.

Swan, Sonnenschein and Co.

"Avenues to Health." By Eustace H. Miles, M.A.

### The Student of Medicine.

### Appointments.

**J. D. Alexander**, L.R.C.P.Edin., L.F.P.S.Glasg., appointed Certifying Factory Surgeon for the Porthcawl District of Glamorgan. **H. A. Bull**, M.R.C.S., L.R.C.P.Lond., appointed Medical Officer for the Colwick District of the Stafford Union. **S. A. Cood**, M.R.C.S., L.R.C.P.Lond., appointed District Medical Officer of the Luton Union. **B. G. Forman**, M.B., Ch.B.Edin., appointed District Medical Officer of the Scarborough Union. **Miss A. Josephine Gardner**, M.B., Ch.B.Edin., appointed Resident Medical Officer to the Bruntsfield Hospital for Women and Children, Edinburgh. **John Harley Gough**, M.D., F.R.C.S.Edin., appointed Admiralty Surgeon and Agent for the Torquay and Babbacombe District. **J. W. G. Grant**, L.R.C.P.Lond., M.R.C.S.Eng., appointed Certifying Factory Surgeon for the Llanwityd Wells District of the County of Brecon. **R. Palk Griffin**, M.R.C.S.Eng., L.R.C.P.Lond., appointed District Medical Officer to the St. Columb Major Union. **H. E. Haycock**, L.R.C.P.Edin., M.R.C.S.Eng., appointed Certifying Factory Surgeon for the Alfreton District of Derbyshire. **H. D. Johns**, M.D., B.S.Dur., appointed Medical Officer to the Hornsey Urban District Council. **Mark McDonald**, M.B., B.Ch.Dub., appointed Certifying Factory Surgeon for the Portaferry District of the County of Down. **R. R. Macnicol**, M.B., Ch.B.Glasg., appointed Certifying Factory Surgeon for the Taynuilt District of Argyllshire. **T. H. Milroy**, M.D., F.R.S.E., appointed Dunville Professor of Physiology, Queen's College, Belfast. **C. F. Myers-Ward**, M.R.C.S., L.R.C.P., appointed Lecturer in Physiology in the Charing Cross Hospital Medical School. **Nathan Raw**, M.D., M.R.C.P.Lond., F.P.S.Edin., D.P.H., appointed Physician to the Sanatorium for Consumption at Haswall, Liverpool. **William Reid**, M.A., M.B., Ch.B.Edin., appointed Junior Assistant Medical Officer to the Burntwood Asylum, Lichfield. **Knowles Renshaw**, M.A., M.D., B.C.Cantab., appointed Assistant Physician to the Manchester Hospital for Consumption and Diseases of the Throat and Chest. **S. F. Crowther Smith**, M.B.Lond., M.R.C.S.Eng., appointed Certifying Factory Surgeon for the Liss District of Hants. **Walter Spencer**, L.R.C.P., L.R.C.S.Edin., appointed Certifying Factory Surgeon for the Bolsover District of Derbyshire. **J. A. Tate**, M.B., B.S., R.U.I., appointed Assistant Medical Officer to the Burnley Union Workhouse. **W. H. Williams**, M.B., C.M.Edin., appointed Medical Officer and Public Vaccinator for the Llanidan (Anglesey) District of the Carnarvon Union.

### "The Hospital" Institutional Library.

"Hospitals and Asylums of the World." 4 Vols., with a Portfolio of Plans, complete £12 12s.
"Burdett's Hospitals and Charities, 1902." 5s.
"Cottage Hospitals, General, Fever, and Convalescent." 10s. 6d.
"Hospital Expenditure: The Commissariat." 2s. 6d.
"A Legal Handbook for the Use of Hospital Authorities." 2s. 6d.
"The Cottage Hospital Case Book or Register of Patients." 10s. and 12s. 6d.
"The Furnishing and Appliances of a Cottage Hospital." 6d.
All these are published by the Scientific Press, Ltd., and may be obtained through any bookseller, or direct from the publishers, 28 and 29 Southampton Street, Strand, London, W.C.

